# Estimated exposure to perfluoroalkyl substances during infancy and serum-adipokine concentrations in later childhood

**DOI:** 10.1038/s41390-023-02665-4

**Published:** 2023-06-14

**Authors:** Philippe Grandjean, Yu-Hsuan Shih, Louise Helskov Jørgensen, Flemming Nielsen, Pál Weihe, Esben Budtz-Jørgensen

**Affiliations:** 1 Department of Environmental Medicine, University of Southern Denmark, Odense, Denmark; 2 Department of Environmental Health, Harvard T.H. Chan School of Public Health, Boston, MA 02115 USA; 3 Department of Biomedical and Pharmaceutical Sciences, University of Rhode Island, Kingston, RI 02881 USA; 4 Department of Clinical Biochemistry, Odense University Hospital and Institute of Clinical Research, University of Southern Denmark, Odense, Denmark; 5 Department of Occupational Medicine and Public Health, Faroese Hospital System, Torshavn, Faroe Islands; 6 Center of Health Science, University of the Faroe Islands, Torshavn, Faroe Islands; 7 Section of Biostatistics, Department of Public Health, University of Copenhagen, Copenhagen, Denmark

## Abstract

**Background:**

Perfluoroalkyl substances (PFASs) are transferred through human milk and may cause elevated exposure during infancy. Given the lack of early postnatal blood samples, PFAS concentrations can be estimated to serve as predictors of subsequent metabolic toxicity.

**Methods:**

A total of 298 children from a prospective birth cohort were followed up through to age 9 years. Serum-PFAS was measured at birth and 18 months of age, while exposures during infancy were estimated by structural equations. Adiponectin, resistin, leptin, and the leptin receptor were measured in serum at age 9. Adjusted regression coefficients for estimated serum-PFAS concentrations were calculated, with additional consideration of the duration of breastfeeding and potential effect modification by sex.

**Results:**

A doubling in estimated serum-PFAS concentrations, particularly at ages 6 and 12 months, was associated with a loss of about 10–15% in age 9 resistin concentrations, while other associations were much weaker. Sex dependence of the associations was not observed, and neither did the duration of breastfeeding affect outcomes at age 9.

**Conclusion:**

Lowered serum-resistin concentrations at age 9 years were most strongly associated with early postnatal PFAS exposures. These findings suggest that infancy may represent a vulnerable time window for some aspects of metabolic programming that may be affected by PFAS exposure.

**Impact:**

- Serum-PFAS concentrations during infancy can be estimated in the absence of blood samples.
- Adipokine concentrations were measured at age 9 years as metabolic biomarkers.
- Resistin was significantly lower in children with elevated PFAS exposures in infancy.
- The findings suggest that early postnatal PFAS exposures may affect subsequent metabolic health.
- Assessment of infancy vulnerability to PFAS can be explored using estimated serum-PFAS concentrations.

## Introduction

Prenatal exposure to a variety of adverse factors, such as maternal smoking, alcohol consumption, maternal dietary deficiency or nutrient oversupply, may affect the later health of the offspring,^1^ and this “fetal programming” may also be affected by toxicity due to environmental chemicals.^2^ Less emphasis has been placed on early postnatal exposures, although the existence of infancy vulnerability is beyond doubt.^3^ Thus, understanding of the temporal profile of toxicant exposures with regard to vulnerable developmental stages is crucial for public health purposes.^4^

Early postnatal exposure can be of particular relevance for substances that are excreted into human milk, such as perfluoroalkyl substances (PFASs).^5,6^ Serum-PFAS concentrations in infants breastfed for 6 or more months may increase by as much as 10-fold at the end of infancy as compared to those not breastfed.^5^ Among adverse health outcomes, immune functions appear to be particularly sensitive to early postnatal exposures.^7^ In addition, obesity seems to be initiated in childhood,^8–10^ although the age-dependent vulnerability to obesogenic or other metabolic effects is unclear. Research in this field is hampered by the frequent lack of blood samples at early ages that could be analyzed for exposure biomarkers. Also, common clinical variables, such as growth trajectories, may not be sufficiently sensitive to reveal early disruptions. Thus, certain metabolic biomarkers in serum, such as adipokines, may be useful as indicators of metabolic homeostasis, inflammation, and the possible risk of developing early signs of metabolic syndrome.^11,12^ These hormonal factors include leptin and its receptor, adiponectin, and resistin, which are associated with childhood growth and with the odds of being or becoming overweight and developing chronic disease.^12–14^ Serum-adipokine concentrations may be affected by other factors, e.g., preterm birth that appears to be associated with lower adiponectin and higher leptin and resistin in later childhood.^14,15^ In addition, the duration of breastfeeding may be of potential importance, as the presence of adipokines has been detected in human milk.^16,17^

Recent studies have begun to explore the possible impact of developmental PFAS exposure and serum concentrations of adipokines measured in later childhood,^18^ with most attention being paid to prenatal PFAS exposure and its associations with leptin and adiponectin.^14,18–22^ We previously observed longitudinal associations between PFAS exposures measured in maternal pregnancy serum or in child serum from age 5 years and serum-adipokine concentrations in childhood.^23^ In agreement with other recent evidence,^14^ we found that adipokine concentrations in cord blood were not associated with prenatal PFAS exposures. On the other hand, serum-adipokine concentrations measured at age 9 years were primarily associated with PFAS concentrations in cord blood and serum from age 18 months and only to a lesser degree with serum-PFAS at 5 and 9 years.^24^ These findings suggest that adipokine concentrations in later childhood may be affected by early-life exposures. As PFAS exposures after birth can increase substantially due to breastfeeding,^5,25^ the aim of the present study was to ascertain the possible association of childhood adipokine concentrations with serum-PFAS concentrations in infancy. Because blood samples were not available from early postnatal age, the PFAS concentrations were modeled by a structural equations approach that has been previously validated.^7^

## Methods

### Study population

The study population is a subset of a birth cohort of 490 mother–child pairs recruited between October 2007 and April 2009 from the National Hospital in Tórshavn, Faroe Islands (Cohort 5). Only singleton, full-term births with complete information were included in the present study. Blood from the cord was collected at parturition, and additional blood samples from the child were obtained at follow-up clinical examinations at ages 18 months, and 5 and 9 years, where questionnaires and physical examinations were also administered.^7,26^ The study protocol was approved by the Faroese ethical review committee and the Harvard T.H. Chan School of Public Health institutional review board. Written informed consent was obtained from all participating mothers.

### PFAS analysis of serum samples

Exposures to five major PFASs (i.e., perfluorooctane sulfonic acid (PFOS), perfluorooctanoic acid (PFOA), perfluorohexane sulfonic acid (PFHxS), perfluorononanoic acid (PFNA) and perfluorodecanoic acid (PFDA)) were assessed from all serum samples using online solid-phase extraction followed by high-pressure liquid chromatography with tandem mass spectrometry,^27^ with extraction conducted using a Thermo Scientific EQuan MAX system (Thermo Scientific, San Jose, CA). High precision was suggested by within-batch and between-batch coefficients of variation at <3% and 5–6%, respectively. For participants with values below the limit of detection (0.03 ng/mL for all PFASs), a value of 0.015 ng/mL was assigned.^24^ The quality was confirmed by regular participation with excellent results in the German-External Quality Assessment Scheme organized by the German Society of Occupational Medicine.

### Adipokine hormones

As previously described,^24^ adipokine hormones were measured in serum using commercial ELISA kits according to the instructions from the manufacturer. The following kits from Biovendor (Brno, Czech Republic) were used: leptin (VEN/RD191001100), soluble leptin receptor (OB-R, VEN/RD194002100), total adiponectin (VEN/RD191023100), and resistin (VEN/RD191016100). For adiponectin, all samples were diluted 1:1200 to obtain measurable levels. We evaluated the performance of the kits using a pooled donor serum obtained from the Department of Clinical Immunology at Odense University Hospital. High-performance accuracy was suggested by the low coefficients of variation of 2.9% for leptin, 2.1% for leptin receptor, 5.3% for adiponectin, and 7.4% for resistin. The results allowed calculation of the free leptin index as the ratio between leptin and leptin receptor concentrations^28^ as well as the adiponectin/leptin ratio.^13,29^

### Covariables

Standard questionnaires were used in the obstetric ward to collect maternal age (years), maternal smoking during pregnancy (none, 1–5 cigarettes per day, >5 cigarettes per day), maternal education (low, medium, high), and child sex. Additional obstetric information was abstracted from hospital charts, including gestational age (weeks), parity (primiparous, multiparous) and pre-pregnancy weight, height, and body mass index (BMI, kg/m^2^). Duration of breastfeeding (months; i.e., exclusive, mixed, and none) was obtained from the 18-month maternal questionnaires.

Potential confounders were selected using directed acyclic graphs based on prior literature on early postnatal PFAS concentrations and childhood metabolic parameters.^8,23,30^ Maternal age, maternal pre-pregnancy BMI, maternal smoking during pregnancy, parity, maternal education, and child sex were considered mandatory for adjustment of all analyses.

### Statistical analyses

PFAS concentrations were log-transformed, and adipokine concentrations were similarly transformed due to skewed distributions. We modeled early postnatal serum-PFAS concentrations at 3, 6, and 12 months based on breastfeeding information as well as the measured PFAS concentrations in cord serum and in the child’s serum at age 18 months.^7^ This was achieved by using structural equation models in an approach as previously described.^7^ In short, the model assumes that the log-transformed PFAS concentration changes by a slope of α during the period of exclusive breastfeeding and likewise, during partial breastfeeding, the slope is *β*, and after weaning, the slope is *γ*:

$$\log {{{{{{\rm{PFAS}}}}}}}_{i,a}=\mu +\alpha \,{{{{{{\rm{exclusive}}}}}}}_{i,a}+\beta \,{{{{{{\rm{partial}}}}}}}_{i,a}+\gamma \,{{{{{{\rm{nomilk}}}}}}}_{i,a}+{U}_{i}+{\varepsilon }_{i,a},,$$

where log PFAS_*i,a*_ is the log-transformed serum concentration of child *i* at age *a*, while exclusive_*i,a*_, partial_*i,a*_, and nomilk_*i,a*_ indicate the number of months that the child was exclusively breastfed, partially breastfed, and not breastfed at all by age *a*. The variable U_*i*_ accounts for within-child correlation, i.e., the fact that a child above the mean at one timepoint will also tend to be higher at other time points. The last term (*ε*_*i,a*_) is a random measurement error. According to the model, the true PFAS concentration of child *i* at age *a* is given by the latent variable *E*_*i,a*_ =*µ* + *α* exclusive_*i,a*_ + *β* partial_*i,a*_ + *γ* nomilk_*i,a*_ + *U*_*i*._. In the second part of the model, this variable was then considered a predictor of the serum-adipokine concentration at age 9 years by linear regression adjusted for covariates. The regression coefficients were expressed as the change for each doubling of the serum-PFAS concentrations at ages 3, 6, and 12 months. Interactions by child sex were assessed by including an interaction term between PFAS and sex in the model. We tested for this interaction (*p*_sex_) based on the corresponding value of the likelihood ratio test statistic. Although the duration of exclusive and mixed breastfeeding was not associated with adipokine hormone concentrations at age 9 years (*p* > 0.1), we included a sensitivity analysis taking into account the duration of breastfeeding.

All regression analyses were conducted using R software, and the structural equation models were fitted using R software’s Lava package.^31^

## Results

The present study included 298 mother–child pairs with complete data on serum-PFAS concentrations at birth and 18 months, adipokine hormones at 9 years, and important covariables; little difference was apparent when compared to the full cohort (Table 1). Most mothers were non-smokers (83%), and approximately equal numbers of boys (52%) and girls (48%) were enrolled. The characteristics differed only slightly from those of the total Cohort 5. The distributions of the serum-PFAS concentrations at birth and 18 months are shown in Table 2. All five PFAS concentrations at 18 months were substantially higher than those at birth, as previously reported,^24^ although 33 infants showed PFHxS at 18 months below the level of detection (and were assumed to be 0.015 ng/mL). The estimated serum-PFAS concentrations during infancy show that a substantial increase occurred during the first postnatal year (Table 2).

**Table 1:** Characteristics of the 298 mother–child pairs, as compared to general cohort.

Characteristics	Study group (*n* = 298)	Total cohort (*N* = 490)
Maternal age, mean ± SD	29.8 ± 5.5	29.7 ± 5.7
Maternal education, *n* (%)		
Low	95 (31.9)	194 (29.5)
Medium	78 (26.1)	247 (37.6)
High	125 (41.9)	215 (32.8)
Maternal smoking, *n* (%)		
No	252 (84.6)	408 (84.1)
1–5 cigarettes per day	28 (9.4)	40 (8.2)
>5 cigarettes per day	18 (6.0)	36 (7.7)
Parity, *n* (%)		
Primiparous	89 (29.9)	147 (30.2)
Multiparous	209 (71.1)	339 (69.8)
Pre-pregnancy BMI, mean ± SD	24.5 ± 4.4	24.3 + 4.3
Child sex, *n* (%)		
Male	153 (51.3)	251 (51.2)
Female	145 (48.7)	239 (48.8)
Duration of exclusive breastfeeding, *n* (%)		
<1 month	34 (11.4)	45 (9.0)
1–3 months	42 (14.1)	59 (11.8)
>3 months	222 (74.5)	349 (79.2)

*SD* standard deviation, *BMI* body mass index.

**Table 2:** Median and interquartile range (IQR) of estimated PFAS concentrations at 3, 6 and 12 months as well as the measured serum-PFAS concentrations at birth and 18 months in the 298 members of the birth cohort.

PFAS (ng/mL)	Birth	3 months	6 months	12 months	18 months
PFOS	2.86 (1.85)	4.04 (2.03)	5.27 (3.02)	6.60 (4.47)	6.89 (5.46)
PFOA	0.89 (0.69)	1.31 (0.70)	1.79 (1.07)	2.40 (1.79)	2.75 (2.49)
PFHxS	0.17 (0.10)	0.25 (0.16)	0.28 (0.19)	0.30 (0.33)	0.23 (0.31)
PFNA	0.31 (0.17)	0.45 (0.19)	0.62 (0.27)	0.88 (0.56)	0.96 (0.75)
PFDA	0.09 (0.05)	0.12 (0.04)	0.15 (0.06)	0.22 (0.11)	0.28 (0.19)

**Fig. 1:**
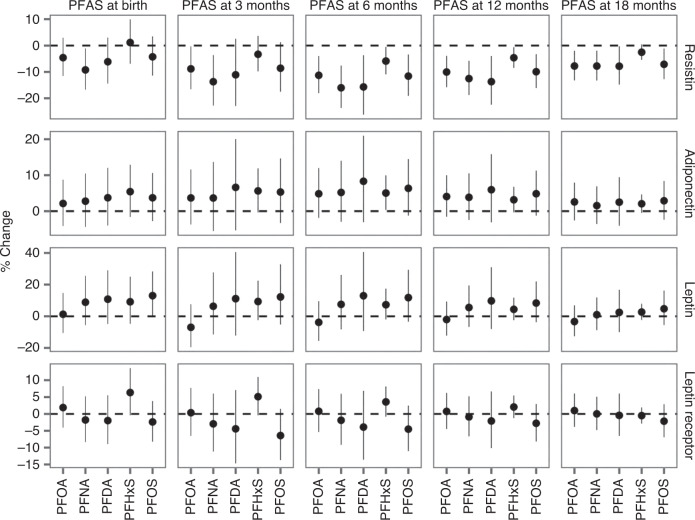
Percent change of serum-adipokine hormone concentrations at age 9 years per doubling of the estimated PFAS concentrations at birth and 3, 6, and 12 months in the overall study population. For comparison, previously reported results^24^ from serum-PFAS at birth and 18 months are also shown.

Associations of the estimated serum-PFAS concentrations at 3, 6, and 12 months with serum-adipokine concentrations at 9 years are shown in Table 3. Figure 1 compares these results with the previously obtained regression coefficients for the serum-PFAS concentrations measured at birth and at 18 months.^24^ For leptin, no significant associations with PFAS exposures were seen (Table 3), as was also the case with the leptin receptor and the free leptin index (Supplementary Table 1). For adiponectin, positive associations were observed for all PFASs, with a statistically significant increase for infancy-age PFHxS (Table 3). Thus, a doubling of PFHxS at age 6 months was associated with an increase of 5.0% in adiponectin at 9 years. Furthermore, the free leptin index (Supplementary Table 2) showed only marginal associations with the estimated PFAS exposures. Likewise, the adiponectin/leptin ratio^15^ was not associated with the exposure parameters. By far, the strongest associations were observed for resistin, where all PFASs showed significant associations during infancy (Table 3), while no clear association was observed with the measured PFOS and PFOA concentrations at age 18 months (Fig. 1). A doubling in late infancy PFAS exposure was associated with resistin decreases of about 10% or more at age 9 years.

**Table 3:** Association of a doubling in estimated serum-PFAS concentration at three different infancy ages with a change (in %) in serum concentrations of leptin, adiponectin, and resistin at 9 years, with confidence intervals (CI) and probability (*p*) values, also for sex interaction in the 298 members of the birth cohort.

		Leptin				Adiponectin				Resistin			
PFAS	Age in months	Change	95% CI	*p*	*p*_sex_	Change	95% CI	*p*	*p*_sex_	Change	95% CI	*p*	*p*_sex_
PFOS	3	12.2	−5.20; 32.8	0.18	0.10	5.31	−3.28; 14.7	0.23	0.68	−8.59	−17.5; 1.29	0.09	0.97
	6	11.8	−3.43; 29.4	0.14	0.17	6.34	−1.22; 14.5	0.10	0.71	**−11.6**	−19.1; -3.39	0.007	0.80
	12	8.38	−3.70; 22.0	0.18	0.37	4.85	−1.22; 11.3	0.12	0.69	**−9.92**	−16.1; -3.26	0.004	0.26
PFOA	3	−6.96	−19.6; 7.60	0.33	0.92	3.67	−3.70; 11.6	0.34	0.65	**−8.82**	−16.6; −0.34	0.04	0.75
	6	−3.82	−15.6; 9.55	0.56	0.89	4.84	−1.85; 12.0	0.16	0.71	**−11.3**	−18.0; −3.98	0.003	0.89
	12	−2.04	−12.2; 9.31	0.71	0.76	4.06	−1.54; 10.0	0.16	0.86	**−10.0**	−15.8; −3.89	0.002	0.54
PFHxS	3	9.32	−2.44; 22.5	0.13	0.41	5.63	−0.31; 11.9	0.06	0.85	−3.30	−9.79; 3.65	0.34	0.41
	6	7.32	−1.96; 17.5	0.13	0.57	**5.04**	0.33; 9.96	0.04	0.90	−**5.88**	−10.9; −0.53	0.032	0.67
	12	4.40	−2.45; 11.7	0.21	0.82	3.16	−0.31; 6.75	0.08	0.98	−**4.61**	−8.47; −0.59	0.025	0.55
PFDA	3	11.1	−12.2; 40.5	0.38	0.23	6.61	−5.32; 20.0	0.29	0.91	−11.1	−23.0; 2.60	0.11	0.66
	6	13.0	−9.28; 40.7	0.28	0.29	8.31	−3.04; 21.0	0.16	0.99	**−15.7**	−26.2; −3.66	0.012	0.77
	12	9.74	−8.06; 31.0	0.30	0.46	5.96	−3.09; 15.8	0.20	0.80	**−13.7**	−22.5; −3.95	0.007	0.49
PFNA	3	6.34	−11.4; 27.7	0.51	0.11	3.66	−5.49; 13.7	0.45	0.89	**−13.7**	−22.8; −3.61	0.009	0.60
	6	7.55	−8.30; 26.1	0.37	0.17	5.20	−2.94; 14.0	0.22	0.99	**−16.0**	−23.7; −7.58	<0.001	0.61
	12	5.56	−6.71; 19.4	0.39	0.37	3.84	−2.44; 10.5	0.24	0.86	**−12.5**	−18.8; −5.80	<0.001	0.17

Bold numbers indicate statistically significant results.

Overall, these tendencies appeared substantially stronger for estimated PFAS exposures during the first year of life than those previously reported for serum-PFAS measured at birth or at later childhood ages.^14, 24^ Thus, although the confidence intervals were wider for the estimated PFAS values (Fig. 1), clear associations were observed for resistin in regard to infancy exposures, while only PFHxS, PFNA and PFDA showed a significant association at 18 months of age, and none at birth or at ages 5 and 9 years. Regarding interaction with sex, no statistical significance was found at infancy ages (Table 3 and Supplementary Fig. 1). In sensitivity analyses that additionally included breastfeeding in the model, only marginal changes were observed (*p* > 0.1).

## Discussion

The present study relied on estimated serum-PFAS concentrations in infancy using a structural equation model using procedures previously described and validated.^7^ As a likely result of continued breastfeeding, PFAS concentrations increased during infancy. Likewise, the serum-PFAS at age 18 months showed higher concentrations than those observed prenatally or at later childhood ages 5 and 9 years,^24^ as shown in Table 2. The elevated exposures during early life were associated with changes in serum-adipokine concentrations at age 9 years, most strongly with reductions in resistin. No clear sex-specific association was observed between early-life exposures to PFAS in relation to the subsequent adipokine outcomes (Table 3 and Supplementary Fig. 1). Our previous study of a subset from the older Faroese Cohort 3—at much higher exposures to PFOS and PFHxS—suggested inverse associations, some being sex-dependent, between PFAS exposures and adiponectin, leptin, and resistin,^23^ but only the latter association was replicated at the lower serum-PFAS concentrations in the new cohort.

Due to the central role of the adipokines, the associations identified are of substantial interest regarding PFAS-associated programming of metabolic disease.^32^ Thus, adiponectin is reduced and leptin is upregulated in the presence of obesity,^11,33^ and resistin tends to be elevated in metabolic disease.^34^ Thus, an imbalance in the expression of the pro- and anti-inflammatory adipokines seems to affect metabolic homeostasis.^12^ Still, considering the possible interactions between adipokines, the PFAS-associated changes in serum concentrations at age 9 are so far difficult to interpret in detail, given the few relevant studies from infancy and childhood. However, the additional observation of decreased leptin in cord blood at elevated PFAS exposures,^22,30^ supports the hypothesis of early-life PFAS exposures influencing these metabolic markers in later childhood. Furthermore, studies in adults indicate that adiponectin concentrations may be affected by PFAS exposures.^35–37^ Thus, the strong associations between estimated infancy exposures to PFAS and the later adipokine concentrations, especially of resistin, support the notion that exposures occurring during early postnatal life may affect important metabolic programming processes.

The presence of adipokines in human milk may suggest that breastfeeding can also affect early metabolic programming,^17^ and for this reason, sensitivity analyses included adjustment for the duration of breastfeeding. No impact was identified, perhaps because adipokine concentrations in milk appear to vary and depend on a multitude of factors. Thus, although the transfer of adipokines via milk has been reported, the duration of breastfeeding seems not to be an important predictor of the child’s serum-adipokine concentrations later on. This issue is of importance, as lactation is a major elimination pathway for maternal PFAS burdens, and duration of exclusive breastfeeding is a key predictor of the child’s early postnatal exposure, particularly for PFOS and PFOA.^5,25^ Thus, the differential patterns of PFAS associations with the adipokines presented in this study likely reflect the changing PFAS exposure profiles early postnatally. Although this is the first study to address PFAS exposures in infancy and their associations with adipokines measured in later childhood, it had to rely on estimated serum-PFAS concentrations, as infancy serum samples were not available. The use of structural equations for this purpose has been previously used to link infancy-age PFAS exposures to deficient antibody responses to childhood vaccines later on.^7^ However, given that the infancy exposures were estimated and not measured, it was not possible to model the total impact of the PFASs and its temporal variation.

As a main finding in this study, the estimated infancy-age PFAS exposures tended to show stronger associations with the adipokine concentrations at age 9 than did the prenatal exposures, as observed most clearly for resistin. This adipokine seems to modulate insulin resistance and is linked to both metabolic and cardiovascular disease.^34^ Still, the PFAS-associated lowering of resistin at age 9 years needs further documentation to allow a more detailed interpretation. The wide confidence intervals for the estimated exposures (Fig. 1) may hide additional associations, and actual PFAS analyses of infant blood samples in the future may well result in better precision. Study protocols for new birth cohorts should therefore consider the possible inclusion of blood sampling at early postnatal ages to obtain more accurate information that may lead to better insight into early-life PFAS exposures and their possible associations with adipokine concentrations and other metabolic markers in later childhood.

A major strength of this study is the prospective study design, which allowed us to examine developmental PFAS exposures in regard to adipokine concentrations in later childhood. Such associations between variables separated in time likely minimize the possibility of reverse causation. Although some adipokine variables failed to show significant associations with the exposure data, the uniformly negative tendencies for resistin strongly support the notion that early postnatal PFAS exposure can impact later childhood adipokine status.

Early-life exposure to PFAS is considered of prime toxicological importance,^2,3^ and the recent guidelines for limiting PFAS exposures in the EU were developed to protect the fetus and the infant as the most vulnerable population, primarily for immunotoxicity.^38^ Given that human resistin is involved, e.g., in the regulation of inflammation,^39^ changes in resistin and perhaps other adipokines may have implications for metabolic patterns as well as immune functions that both deserve attention concerning possible early postnatal (re)programming affected by PFAS exposure.

## Conclusions

In 298 children, clear, negative associations were found for their serum-resistin concentrations at age 9 years in regard to estimated infancy-age PFAS exposures; in addition, weak, positive associations were observed for the age 9 adiponectin concentration. These associations tended to be stronger than those observed with prenatal PFAS exposures; sex dependence of the associations was not observed. These findings suggest that infancy may represent a vulnerable time window for certain forms of PFAS-associated metabolic programming.

### Disclaimer

The authors are solely responsible for all results and conclusions, which do not necessarily reflect the official position of the National Institute of Environmental Health Sciences of the NIH or any other funding agency. The use of trade names is for identification only and does not imply any endorsement.

### Supplementary Information

Supplementary Information

Supplementary Figure

## Data Availability

The dataset analyzed in this study is not publicly available due to national data security legislation on sensitive personal data.
