# Supplementary material for: Estimated exposure to perfluoroalkyl substances during infancy and serum-adipokine concentrations in later childhood
Source: Pediatr Res. 2023 Jun 14;94(5):1832–7. doi: 10.1038/s41390-023-02665-4 (PMC10624607; doi:10.1038/s41390-023-02665-4)
Supplement: Supplementary file 1 — Supplementary Information [file 41390_2023_2665_MOESM1_ESM.pdf]

Supplemental

Supplemental Table 1. Association of a doubling in serum-PFAS concentration at three different infancy ages with a change (in %) in *leptin receptor* and the *leptin/leptin receptor ratio* concentration at 9 years, with confidence intervals (CI) and probability (p) values also for sex interaction.

		Leptin receptor				Leptin/leptin receptor ratio			
Exposure	Age (months)	Change	95% CI	*p*	P_{sex}	Change	95% CI	*p*	P_{sex}
	3	0.36	-8.14 ; 6.48	0.92	0.07	-8.28	-24.68 ; 11.70	0.390	0.476
PFOA	6	0.79	-6.33 ; 6.75	0.81	0.10	-5.80	-20.88 ; 12.16	0.502	0.577
	12	0.75	-5.14 ; 5.88	0.78	0.16	-3.73	-16.88 ; 11.49	0.611	0.756
	3	-2.95	-11.2 ; 6.05	0.51	0.47	9.34	-13.54 ; 38.28	0.456	0.060
PFNA	6	-1.88	-9.18 ; 6.00	0.63	0.52	8.71	-11.44 ; 33.44	0.425	0.106
	12	-0.88	-6.65 ; 5.24	0.77	0.56	5.85	-9.99 ; 24.48	0.492	0.247
	3	-4.42	-14.7 ; 7.09	0.44	0.13	13.48	-13.01 ; 48.05	0.351	0.052
PFDA	6	-3.91	-13.6 ; 6.86	0.46	0.14	14.10	-11.26 ; 46.71	0.304	0.066
	12	-2.09	-10.1 ; 6.68	0.63	0.20	10.41	-10.85 ; 36.73	0.364	0.134
	3	5.13	-0.40 ; 11.0	0.07	0.25	5.60	-6.48 ; 19.25	0.379	0.018
PFHxS	6	3.57	-0.82 ; 8.16	0.11	0.35	4.22	-5.77 ; 15.25	0.422	0.069
	12	2.06	-1.23 ; 5.46	0.22	0.46	2.80	-5.37 ; 11.66	0.513	0.195
	3	-6.42	-13.7 ; 1.50	0.11	0.11	22.10	-2.40 ; 52.74	0.081	0.013
PFOS	6	-4.52	-11.0 ; 2.48	0.20	0.18	17.74	-2.97 ; 42.86	0.098	0.042
	12	-2.78	-8.19 ; 2.95	0.34	0.26	11.76	-4.63 ; 30.96	0.169	0.133

LEGEND

Supplemental Figure 1. Percent change of serum-adipokine hormone concentrations at age 9 years per doubling of the estimated PFAS concentrations at birth and 3, 6, and 12 months in males (green) and females (orange). For comparison, results from serum-PFAS at birth and 18 months[1] are also shown.

1 Shih, Y. H., Blomberg, A. J., Jorgensen, L. H., Weihe, P. & Grandjean, P. Early-Life Exposure to Perfluoroalkyl Substances in Relation to Serum Adipokines in a Longitudinal Birth Cohort. *Environ Res* **204**, 111905 (2022).
